# Transcriptomic insights into the blue light-induced female floral sex expression in cucumber (*Cucumis sativus* L.)

**DOI:** 10.1038/s41598-018-32632-7

**Published:** 2018-09-24

**Authors:** Yong Zhou, Golam Jalal Ahammed, Qiang Wang, Chaoqun Wu, Chunpeng Wan, Youxin Yang

**Affiliations:** 1 Jiangxi Key Laboratory of Crop Physiology, Ecology and Genetic Breeding, College of Agronomy/College of Science, Jiangxi Agricultural University, Nanchang, 330045 China; 2 College of Forestry, Henan University of Science and Technology, Luoyang, 471023 China

## Abstract

In cucurbitaceous crops, sex differentiation of flower buds is a crucial developmental process that directly affects fruit yield. Here we showed that the induction of female flower was the highest in the blue light-treated monoecious cucumber plants compared with that in other light qualities (white, green and red). High-throughput RNA-Seq analysis of the shoot apexes identified a total of 74 differently-expressed genes (DEGs), in which 52 up-regulated and 22 down-regulated under the blue light compared with that in white light. The DEGs were mainly involved in metabolic pathways, biosynthesis of secondary metabolites, plant hormone signal transduction, starch and sucrose metabolism and phenylpropanoid biosynthesis. While the  ethylene and gibberellins synthesis and signaling related genes were down-regulated, the abscisic acid and auxin signal transduction pathways were up-regulated by the blue light treatment. Furthermore, the blue light treatment up-regulated the transcription of genes relating to photosynthesis, starch and sucrose metabolism. Meanwhile, the blue light suppressed the GA_3_ concentration but promoted the concentrations of auxin and photosynthetic pigments. Taken together, the results suggest that the blue light-induced female floral sex expression is closely associated with the blue light-induced changes in abscisic acid, auxin, gibberellins, photosynthesis, starch and sucrose metabolism pathways, which is potentially different from the traditional ethylene-dependent pathway.

## Introduction

Sex differentiation is an important plant developmental process mediated by the selective arrest of either the male stamen or female carpel during the flower development. The process has been extensively studied in a range of plant species, including cucurbitaceous vegetables^1^. Particularly, monoecious cucumber (*Cucumis sativus* L.) plants that produce distinct male and female flowers on the same plant, have been served as an ideal model organism to study the sex expression in flowering plants^2,3^. Three major gene loci related to 1-aminocyclopropane-1-carboxylate synthase (*ACS*) in ethylene (ET) biosynthesis pathway have been shown to control sex determination in cucumber. These gene loci are generally called as *female* (*F*)^4,5^, *monoecious* (*M*)^6^, and *androecious* (*A*)^7^, and the combination and interaction of *F*, *M*, and *A* genes eventually determine various sexual phenotypes in cucumber.

In addition to genetic control, plant hormones, especially ET and gibberellin (GA), profoundly affect flowering process in cucumber. ET is considered as the basic ‘sex hormone’ that enhances the female tendency in monoecious cucumber, whereas gynoecious genotypes produce an increased level of ET compared to that of monoecious plants^3,8^. Furthermore, ET differentially regulates two sex-related developmental processes, namely sex expression and sex determination^9^. ET perception leads to female flower development in cucumber through the induction of DNA damage^10^. In addition to the *F*, *M*, and *A* genes, other genes involved in either ET biosynthesis or signaling pathways, such as *CsACO2*^8,11^ and *CsETR1*^10^, also play important regulatory roles in sex determination of cucumber. On the other hand, GAs promote stamen and anther development; however, GAs-enhanced male flower formation can be mediated both via ET-dependent and ET-independent pathways in cucumber^12,13^. For instance, exogenous application of GA_3_ enhances formation of the male flowers in gynoecious plants by decreasing ET production, suggesting an antagonistic role of ET and GA in sex expression of cucumber^12^. However, a recent study showed that the biologically inactive precursor GA_9_ can move from ovaries to sepal/petal tissues and convert into bioactive GA_4_, which is necessary for the female flower development in cucumber^14^.

In addition to genetic control and plant hormones, sex expression in cucumber can also be regulated by environmental factors, such as temperature, photoperiod, and light intensity^15–19^. As a signal and energy source, light functions as an essential environment cue that regulates plant reproductive development including sex expression. In general, short days and low light intensity contribute to the female floral sex expression, whereas long days and high light intensity promote induction of the male flowers^18,19^. In addition to light intensity and photoperiod, specific light quality such as blue (B) light can also influence a series of morphological and physiological processes in plants including phototropism, hypocotyl elongation, leaf morphology, chlorophyll fluorescence, stomatal movement, leaf photosynthesis, and genes expression^20,21^. However, little is known about the blue light-regulated sex expression in plants, especially in cucumber.

To understand how blue light regulates the sex expression of cucumber, we compared the effect of blue light and white light on cucumber sex expression by using transcriptome profiles. Our results showed that blue light suppresses ET and GA biosynthetic gene expression and decreases GA content. However, the genes involved in photosynthesis, starch and sucrose metabolism pathways were significantly up-regulated by blue light treatment. Moreover, our results suggest a regulatory involvement of transcription factor MYB in blue light-induced female floral sex expression in cucumber. The results of this study shed new light on light quality-regulated cucumber sex expression, which might have important application in cucumber cultivation under controlled light environments.

## Results

### Female flower formation in response to different light qualities in cucumber

To determine the effect of different light qualities on sex expression of cucumber, we examine the percentage of nodes with female flowers of cucumber under monochromatic such as blue (B), green (G), red (R), and white light (W). A total of 20 nodes from each plant on the main stem were investigated. As shown in Table 1, B-light promoted femaleness with a significant increase in the number of female flowers and caused a significant decrease in node position of the first female flower compared with W, G and R. These results suggest that blue light treatment can increase numbers of female flowers, leading to a significant change in the sex expression of flowers in cucumber.

**Table 1:** Effects of different light qualities on sex expression of cucumber.

Treatments	First node of female flower	Percentage of nodes with female flowers (%)
White light	7.83 ± 0.47^b^	20.70 ± 3.25^b^
Blue light	5.25 ± 0.25^c^	30.15 ± 2.48^a^
Green light	10.73 ± 0.79^a^	15.15 ± 0.78^c^
Red light	10.50 ± 0.49^a^	20.12 ± 1.78^bc^

Note: Cucumber plants were exposed to different lights (W, B, G, and R) for 20 days. The data are the mean ± SD of four replicates with 10 plants as a replicate. Means denoted by the same letter did not significantly differ at *p*  < 0.05 according to Duncan’s multiple range test.

### Effects of blue light on the transcription of GA- and ET-related genes

**Figure 1:**
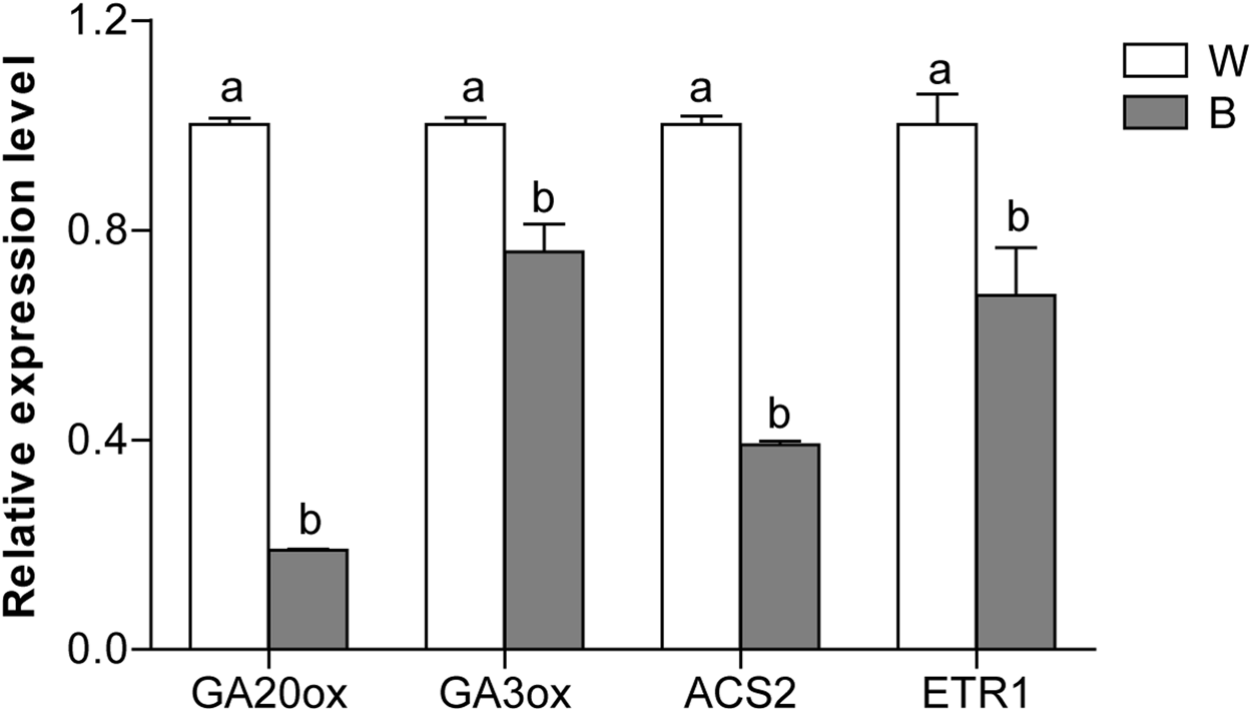
Effect of blue light (B) and white light (W) on the expression of gibberellins biosynthesis (GA20ox and GA3ox), and ethylene biosynthesis (*ACS2*) and signaling (*ETR1*) genes in cucumber shoot apex. Cucumber plants were exposed to B and W light qualities for 20 days until the plants attained the 4-leaf stage. The shoot apexes containing immature leaves shorter than 2 cm in length were sampled and used for qRT-PCR analysis. Data are the average of three biological replicates and are presented as the mean ± SD. Means denoted by the different letters are significantly different at *P* < 0.05, according to Duncan’s multiple range test.

To examine whether the blue light-induced female floral sex differentiation in cucumber was associated with the GA and ET biosynthesis and signaling pathways, the expression levels of *CsGA20ox* and *CsGA3ox*, *CsACS2*, *CsETR1* were analyzed by qRT-PCR. As shown in Fig. 1, these genes were down-regulated in blue-light exposure treatment. The B-reduced the transcript levels of *CsGA20ox*, *CsGA3ox*, *CsACS2* and *CsETR1* by 0.19, 0.76, 0.39 and 0.09-fold compared to W (control), respectively.

### Illumina sequencing and *de novo* assembly

To assess how blue light treatment contributes to the femaleness of cucumber flowers, we performed RNA-Seq analysis using cucumber shoot apex samples treated with W and B. Each treatments contained three biological replicates, and thus six libraries were sequenced. A total of 65,438, 628/65,755,474/47,082,830 (Control, W1/2/3), and 53,642,214/54,185,906/54,056,084 (B1/2/3) raw reads were generated (Table 2, Supplementary Fig. S1). After removal of adaptor sequences, duplication sequences, ambiguous reads and low-quality reads, 328,682,038 high-quality clean reads with a total of 49.29 GB bases remained. Among these clean reads, the average percentage of Q20 (base quality more than 20) was 96.84%, and the GC was 43.86% (Table 2). Each library that produced the clean reads was aligned to the cucumber reference genome, a total of 303,282,572 uniquely mapped clean reads (92.28% of total clean reads) from RNA-Seq data in the six libraries were mapped and uniquely mapped to the cucumber genome, respectively (Table 2).

**Table 2:** Summary of the Illumina transcriptome sequencing and assembly.

Sample name	Raw reads	Clean reads	Clean bases	Q20 (%)	GC content (%)	Total mapped clean reads (%)	Uniquely mapped clean reads (%)
W1	65,438,628	63,631,440	9.54	97.03	43.90	59666025 (93.77%)	58522148 (91.97%)
W2	65,755,474	63,083,108	9.46	97.14	44.02	59184179 (93.82%)	58037416 (92.00%)
W3	47,082,830	45,625,738	6.84	96.16	43.92	42692294 (93.57%)	41928406 (91.90%)
B1	53,642,214	51,763,026	7.76	96.56	43.71	48,658,620 (94.00%)	47809329 (92.36%)
B2	54,185,906	51,925,182	7.79	97.18	43.95	48,751,083 (93.89%)	47815191 (92.08%)
B3	54,056,084	52,653,544	7.9	96.97	43.71	50,095,667 (95.14%)	49170082 (93.38%)
Total	340,161,136	328,682,038 (96.62%)	49.29	96.84% (avg)	43.86% (avg)	309,047,868 (94.03%)	303,282,572 (92.28%)

All sequencing raw data in the present study were deposited into the BIG Data Center GSA database under accession numbers CRA000867. All these uniquely mapped reads were considered for further analysis. A list of statistics of genes in different expression-level interval is shown in Table 3. Genes with FPKMs beyond 60 were considered to be expressed at high level, accounting for an average of 33.86% and 11.07%. Genes with FPKMs in the interval 0–1 were considered to be expressed at very low levels or not to be expressed, respectively.

**Table 3:** Statistics of genes in different expression-level interval.

FPKM Interval	W1	W2	W3	B1	B2	B3
0~1	3736 (18.73%)^a^	3574 (17.92%)	3622 (18.16%)	3553 (17.82%)	3594 (18.02%)	3535 (17.73%)
1~3	1711 (8.58%)	1745 (8.75%)	1713 (8.59%)	1768 (8.87%)	1774 (8.90%)	1747 (8.76%)
3~15	5608 (28.12%)	5646 (28.31%)	5605 (28.11%)	5761 (28.89%)	5676 (28.46%)	5528 (27.72%)
15~60	6713 (33.66%)	6766 (33.93%)	6813 (34.16%)	6627 (33.23%)	6699 (33.59%)	6900 (34.60%)
>60	2174 (10.90%)	2211 (11.0%)	2189 (10.98)	2233 (11.20%)	2199 (11.03%)	2232 (11.19%)

Note: W1-3 samples were white light treated samples, B1-3 were blue-light treated samples. FPKM: fragments per kb per million reads. a. Ratios of gene number to total gene number are presented in parentheses.

### Identification of differentially expressed genes (DEGs) and qRT-PCR confirmation

**Figure 2:**
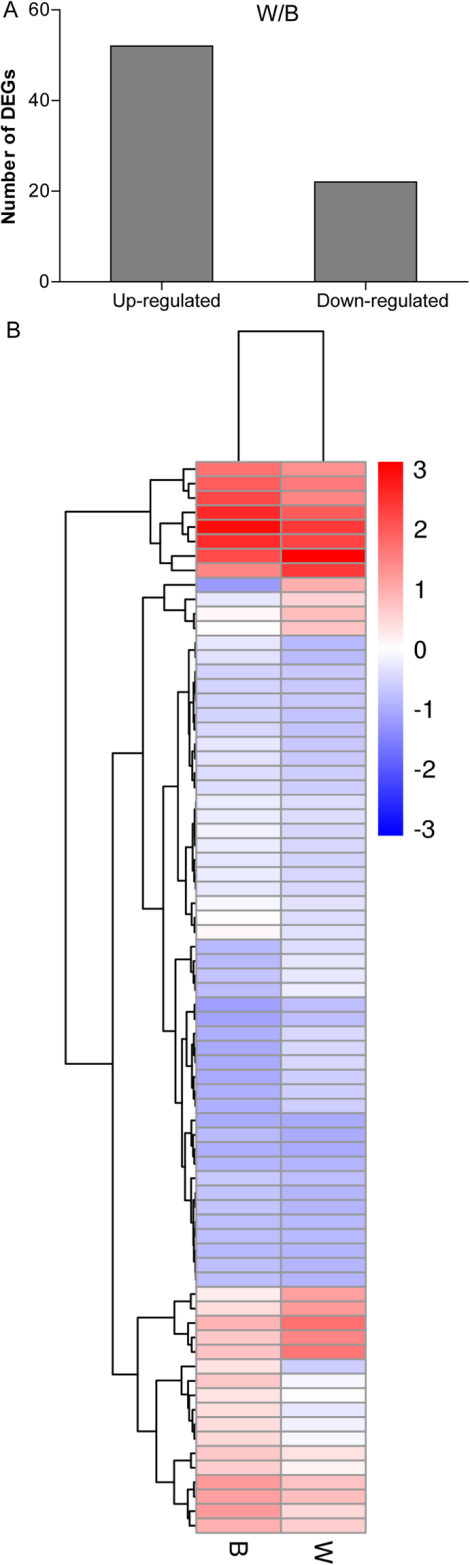
Transcriptome analysis of DEGs under B and W treatments of cucumber. (**A**) Number of DEGs between two different treatments. The *P*-value < 0.05 was used as thresholds to determine the significance of DEGs. (**B**) Hierarchical clustering of DEGs based on the three sample’s log_2_FPKM. The color (from blue to red) represents gene expression intensity from low to high, meaning that blue bands identify low gene expression quantity, and the red ones represent the high gene expression quantity.

Using *P*-value ≤ 0.05 and the absolute value of log_2_FPKM ≥ 1 as the significance cut-offs, we identified 74 DEGs including 52 up-regulated genes and 22 down-regulated genes under B treatment compared with the control (W) (Fig. 2A, Supplementary Table S1). We made a hierarchical clustering of the differentially expressed genes based on the three sample’s log_2_FPKM, so that we could observe the overall gene expression pattern. The blue bands identify low gene expression quantity, and the red ones represent high gene expression quantity (Fig. 2B).

**Figure 3:**
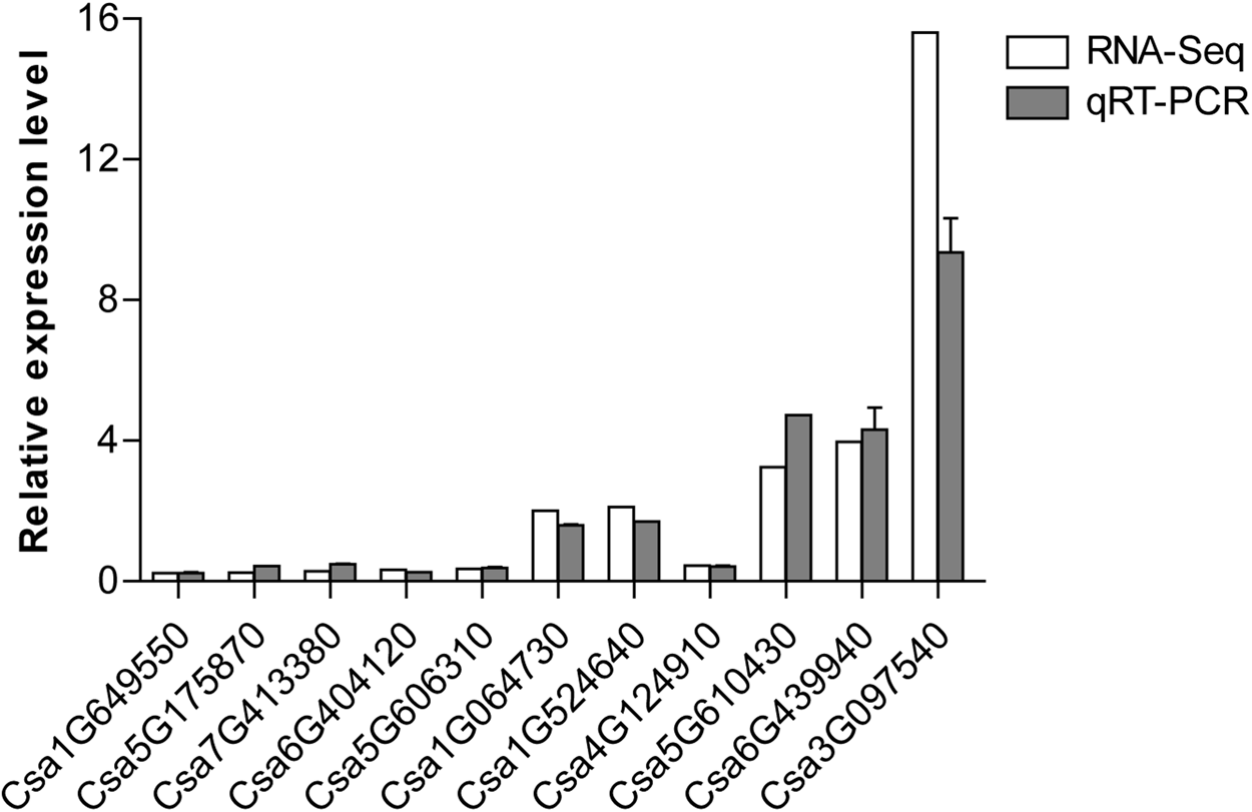
qRT-PCR validation of DEGs identified by RNA-Seq. 11 DEGs including 5 up-regulated genes and 6 down-regulated genes were selected for qRT-PCR confirmation.

To validate the RNA-Seq data, 11 DEGs were randomly selected to analyze their expression by qRT-PCR. The results of the qRT-PCR analysis showed similar trends compared to those obtained by RNA-Seq (r = 0.9499; *P* < 0.0001, Fig. 3 and Supplementary Table S2), indicating that the changes in gene expression detected via RNA-Seq were accurate and thus confirming the reliability of the RNA-Seq data.

### Gene ontology (GO) analysis of DEGs

GO assignments were applied to classify functions of DEGs. All the DEGs were grouped into three major functional categories, including biological process, cellular component, and molecular function, which were further classified into 13, 13, and 7 subcategories, respectively (Supplementary Fig. S2). Genes involved in metabolic process (GO:0008152; 58 transcripts), cellular process (GO:0009987, 31 transcripts), catalytic activity process (GO:0003824, 22 transcripts), single-organism process (GO:0044699, 19 transcripts), and binding (GO:0005488, 19 transcripts) were most highly represented. Massively up-regulated genes were enriched in the GO terms including oxidoreductase activity, oxidation-reduction process, sucrose alpha-glucosidase activity, and chlorophyllide a oxygenase activity. Whereas numerous down-regulated genes were enriched in oxidation-reduction process, oxidoreductase activity, carbohydrate metabolic process, and beta-glucan metabolic process.

### Pathway enrichment analysis for DEGs

**Figure 4:**
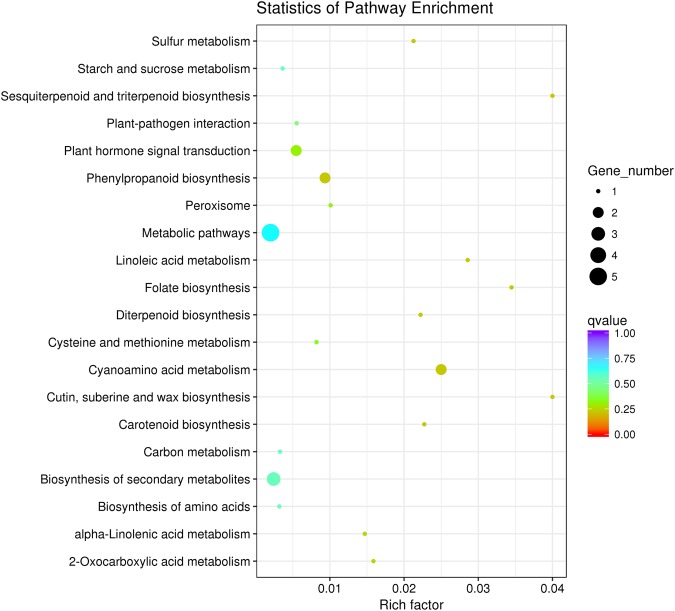
KEGG pathway enrichment analysis of DEGs between W and B treatments. The left Y-axis shows the KEGG pathway. The X-axis shows the rich factor. A high *q*-value is represented by blue and a low q-value is represented by red (*q* < 0.05).

All the DEGs were mapped to KEGG database terms and compared with the whole transcriptome data. These genes were significantly enriched in 20 KEGG pathways (Fig. 4). Those pathways with the greatest representation by DEGs were the metabolic pathways (csv01100) with 5 members and biosynthesis of secondary metabolites (csv01110) with 3 members. It can be also detected that plant hormone signal transduction, starch and sucrose metabolism, carbon metabolism, phenylpropanoid biosynthesis and peroxisome were also significantly enriched (Fig. 4), which implicated that those pathways were involved in the blue light-induced female floral expression. Sesquiterpenoid and triterpenoid biosynthesis was the most enriched pathway in the up-regulated genes, which may play important role in controlling sex expression.

### Transcriptome profiles of hormone-related genes

**Figure 5:**
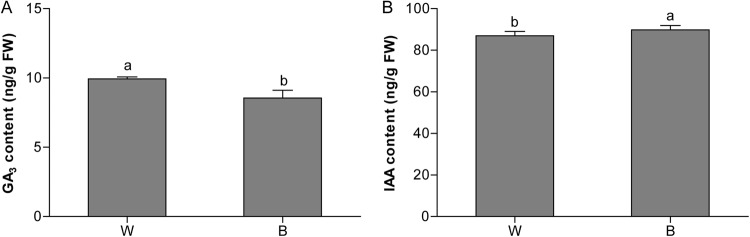
Changes in GA_3_ and IAA content as influenced by the blue (**B**) and white (W, the control) light treatments. Data are the average of three replicates and are presented as the mean ± SD. Means denoted by the different letters are significantly different at *P* < 0.05, according to Duncan’s multiple range test.

Sex expression in cucumber can be affected by different hormones. To investigate the expression of hormone-related genes, BL-regulated sex expression was investigated in cucumber. We first examine the transcriptome profiles of hormone-related genes. As shown in Table 4, several DEGs involved in ET, GA, auxin, and ABA signaling pathways were detected. We found that the expression of an ET-responsive transcription factors (*CsESR2-like*, Csa5G598600) dramatically decreased in B treatment compared to W, while the transcript of *CsACO4* (Csa1G064730) encoding an ACC oxidase increased in B treatment compared to W. In addition, two genes encoding GA biosynthetic enzymes in a series of oxidation steps, Csa6G351370 and Csa7G413380, showed an opposed expression under B treatment (Table 4). We then examine the level of GA_3_ in cucumber shoot apex. The results showed that the GA_3_ content relatively decreased under B compared to that in the control (W) (Fig. 5). Moreover, genes involved in auxin pathway, auxin-induced SAUR-like protein (Csa7G009100), auxin-responsive protein and *IAA32* (Csa5G610430) were highly up-regulated by B light compared with W light. Similarly, as shown in Fig. 5, IAA content also increased under B light. ABA signaling related genes, such as *Abscisic acid 8′-hydroxylases* (Csa2G361840, Csa1G524640) were also up-regulated by B (Table 4). These results indicate that hormones may play crucial roles in BL-regulated sex expression in cucumber.

**Table 4:** List of selected hormone biosynthesis and signaling factors in the DEGs under blue light treatment in cucumber.

Locus ID	Description	Fold change	*P*-value
Csa6G351370	Gibberellin 20-oxidase, putative	2.27	0.05
Csa7G413380	Gibberellin 2-β-dioxygenase 2	0.29	0.01
Csa5G598600	Ethylene-responsive transcription factor	0.49	0.03
Csa7G009100	Auxin-induced SAUR-like protein	3.05	0.03
Csa5G610430	Auxin-responsive protein, IAA32	3.25	0
Csa2G361840	Abscisic acid 8′-hydroxylase	2.07	0.01
Csa1G524640	Abscisic acid 8′-hydroxylase	2.11	0

### Transcriptome profiles of photosynthesis-related genes

**Figure 6:**
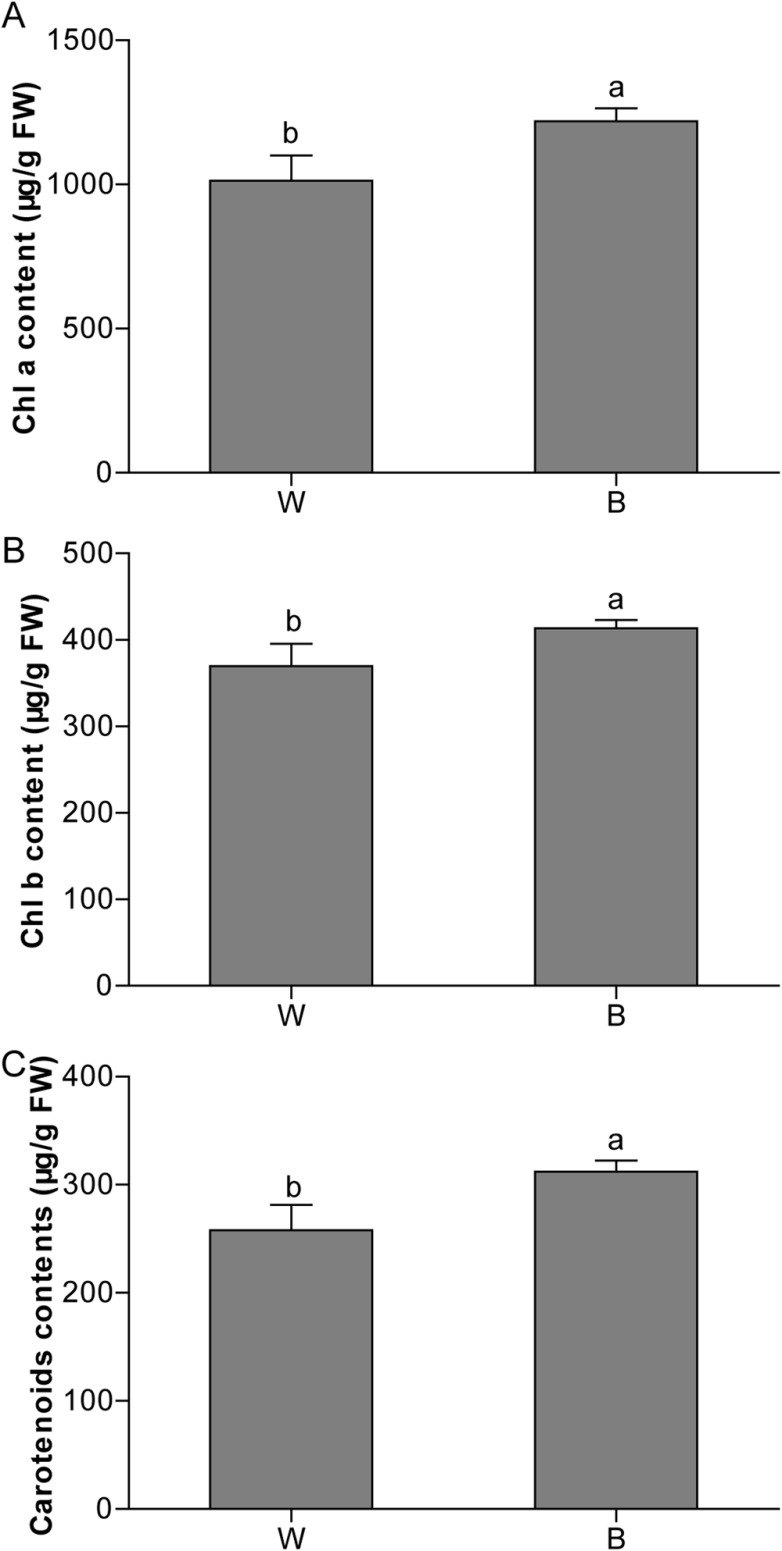
Effects of blue (**B**) and white (W, the control) on the chlorophyll contents in cucumber leaves after 20 d of respective treatments. Data are the average of three replicates and are presented as the mean ± SD. Means denoted by the different letters are significantly different at *P* < 0.05, according to Duncan’s multiple range test.

A total of 5 genes involved in the photosynthesis were up-regulated by the B treatment (Table 5). Among these genes, Csa6G504720 and Csa1G618390 are involved in chlorophyll biosynthesis pathway, implying that cucumber plants grown under monochromatic B had an altered chlorophyll contents compared with plants grown under W. In addition, an early light induced protein, Csa3G145780, was also up-regulated by B treatment (Table 5). To further confirm whether B-induced transcriptional changes altered photosynthetic pigment contents, we analyzed the concentrations of Chl a, Chl b and carotenoids. Results showed that the B-treatment significantly increased Chl a, Chl b and carotenoids concentration compared to that in W-treated seedlings (Fig. 6). These results suggest that B might have a positive role in the photosynthetic processes.

**Table 5:** List of selected photosynthesis-related DEGs under blue light treatment in cucumber.

Locus ID	Description	Fold changes	*P*-value
Csa3G145780	Putative early light induced protein 2	2.71	0.01
Csa1G618390	Isoleucine N-monooxygenase 1-like	6.54	0.00
Csa6G504720	Cytochrome P450	2.53	0.04
Csa5G577430	CHUP1 Protein	2.13	0.05
Csa1G600130	Pheophorbide A oxygenase, putative	2.22	0.02

### Transcriptome profiles of signal transduction-related DEGs

We also analyzed the transcriptome profiles of DEGs relating to signal transduction including some transcription factors and protein kinases. We found that 6 different DEGs from 5 transcription factors families such as *MYB*, *NAC*, *LOB*, *MADS* and zinc finger protein, were differentially expressed by the blue light treatment. A member of *MYB* transcription factors, Csa7G170600, declined under B treatment (Table 6), Whereas another transcription factor, *CsNAC51*, which is an orthologue of the known stress-responsive *ANAC029*/*AtNAP* in *Arabidopsis*, also down-regulated under B treatment (Table 6). *AtNAP* can mediate ABA-regulated stomatal movement and water loss specifically during leaf senescence, and has an important role in fruit senescence^22,23^. Moreover, N deprivation, salinity and ABA treatment can also up-regulate the expression of *CsNAC51* in cucumber^24,25^. These imply that blue light-regulated sex expression in cucumber may be associated with the regulation of stress response genes. In addition, the zinc finger protein CONSTANS-LIKE 2-like (Csa4G124910) was up-regulated, while the zinc finger protein CONSTANS-LIKE 5 (Csa2G057080) was down-regulated by B treatment. However, an Agamous-like MADS-box protein of the *C-class MADS* family gene, Csa6G095280, was down-regulated by B treatment (Table 6). Five genes related to *protein kinase* were differentially expressed by B treatment, among them 4 genes (Csa6G439940, Csa5G517150, Csa1G065390, and Csa5G605030) were up-regulated, while Csa7G452270 was down-regulated.

**Table 6:** List of selected signal transduction-related DEGs under blue light treatment in cucumber.

Locus ID	Description	Fold changes	*P*-value
Csa7G170600	MYB transcription factor	0.43	0.04
Csa5G606310	NAC transcription factor 29-like, CsNAC51	0.35	0.00
Csa5G175870	LOB domain-containing protein	0.25	0.04
Csa4G124910	zinc finger protein CONSTANS-LIKE 2-like, hd1	0.45	0.00
Csa2G057080	zinc finger protein CONSTANS-LIKE 5	2.00	0.00
Csa6G095280	Agamous-like MADS-box protein	0.39	0.00
Csa6G439940	Calcium-dependent protein kinase	3.97	0.00
Csa5G517150	Receptor-like protein kinase	3.05	0.02
Csa1G065390	Cysteine-rich receptor-like protein kinase 7	4.29	0.00
Csa5G605030	PHYTOCHROME KINASE SUBSTRATE 1-like protein	6.06	0.01
Csa7G452270	Leucine-rich repeat receptor-like protein kinase At2g19210	0.47	0.01
Csa5G605030	Putative phytochrome kinase substrate	6.06	0.01
Csa3G097540	Sesquiterpene synthase	15.56	0.00
Csa7G351890	Quinone oxidoreductase, NAD(P)H	3.49	0.00
Csa3G829250	Peroxidase	0.45	0.02

It is worth mentioning that the most highly up-regulated gene *sesquiterpene synthase* (Csa3G097540) is florally expressed and its expression occurs in stigma, nectarines, sepals, and anthers^26^. This gene has also been shown to play important role in sexual development. In addition, putative phytochrome kinase substrate (*PKS*, Csa5G605030) may also control auxin homeostasis and thus integrates cucumber sex expression^27^.

## Discussion

Light is one of the major environmental factors that influence plant growth and development, and different light qualities can lead to different photosynthetic and morphogenetic responses in different plant species^20,21,28^. Despite extensive studies on the mechanisms of endogenous signal (*e.g*. phytohormones)-mediated floral sex expression in plants, our understanding of the environmental signals such as light quality-regulated sex expression in cucumber remains fragmentary. In this study, we found that blue light treatment resulted in an improved percentage of nodes with female flowers, leading to a significant change in the sex expression of flowers in cucumber (Table 1). Since cucumber yield largely depends on the female floral sex expression, the modulation of light environment with the blue light quality from LED could be an efficient way to raise the cucumber yield in protected horticulture.

Previous studies have revealed that phytohormones can affect the sex expression in cucumber^9,12^. Similarly, we found that B-induced alteration in floral sex expression was associated with the changes in the endogenous levels of some key hormones, such as GA_3_ and auxin. In addition, under B treatment, several genes involved in ET, GA, auxin, and ABA signaling pathways were differentially expressed (Fig. 1 and Table 4). Gibberellins play important roles in stamen and anther development in hermaphroditic plants^13,29^, and inhibit the female tendency in cucumber^12^. In addition, GA could mediate sex expression of cucumber via both ET-dependent and ET-independent pathways^12^. In this study, two genes involved in GA biosynthetic pathway were down-regulated by B treatment compared with that of white light (Fig. 1 and Table 4), which was consistent with the decrease in GA_3_ content under B compared to the control (W) (Fig. 5). This implies that the BL treatment might promote female flowers formation by inhibiting GA in the cucumber. In addition, B-induced reduction in GA levels may also inhibit the male flower development and/or promote the female flower differentiation.

According to our qRT-PCR and RNA-Seq results, the expression of ET biosynthesis gene (CsACS2), ET-responsive transcription factor *CsETR1* (Csa2G070880), and *CsERF* (Csa5G598600) was dramatically decreased by B treatment compared to W (Fig. 1, Table 4). ERFs usually act as positive regulators and are involved in ET signal transduction pathway^30^. The expression levels of some *CsERFs* significantly decreased after GA_3_ treatment, indicating a potential involvement of GAs in cucumber sex expression^12^. A recent study showed that auxin-related genes were involved in sex expression of cucumber^31^. Auxin can regulate sex determination indirectly through the modulation of secondary auxin-responsive genes^32^. In the current study, the expression of genes involved in auxin signal pathway was significantly increased by B treatment (Table 4), implying that B treatment might enhance the auxin signaling. In addition, two ABA-signaling pathway genes, Csa2G361840 and Csa1G524640, which encode *ABA 8′-hydroxylase*, were also found to be up-regulated by B treatment (Table 4), implying that ABA synthesis was enhanced by B treatment. These results indicated that auxin and ABA may play particular roles in BL-regulated sex expression of cucumber. Hence, we speculate that blue light-induced female floral sex expression is mediated mainly via decreased GA accumulation and its coordinated interaction with other hormones in cucumber.

Light quality may change the activity of photoreceptors that are involved in signaling and control of plant growth and development. In addition, the efficiency of chlorophylls and carotenoids to capture photons and transfer energy might be altered under selected light wavelengths^20,33^. In this study, we found that Chl a, Chl b and carotenoid levels were significantly higher in the BL-treated cucumber seedlings than those in control (Table 5). The B-increased Chl a, Chl b and carotenoid levels could potentially increase light absorption and decrease photoinhibition, resulting in an increased photosynthetic capacity^20,33^. Our results are consistent with a previous study that exposure of cucumber plants to different percentages of blue and red light using LEDs enhanced leaf photosynthetic capacity, net photosynthetic rate, stomatal conductance, and chlorophyll content with the increase in blue and red-light percentage up to a ratio of 50%:50% (blue light: red light) treatment^21^. A recent study also showed that cucumber plants under the blue light treatment had an increased leaf net photosynthetic rate and stomatal conductance compared with R supplemented with B^33^. In this study, a chloroplast outer envelope protein chloroplast unusual positioning 1 (CHUP1), which is essential for chloroplast anchorage to the plasma membrane and participates in chloroplast relocation movement to reduce photodamage in plants^34,35^, was up-regulated by B treatment (Table 5). In addition, the transcript levels of photosynthesis-related genes remained up-regulated under B treatment (Table 5), indicating that B-induced potential improvement in photosynthesis might increase sugars, such as glucose and sucrose production. This speculation can also be supported by the GO enrichment data that showed that B treatment significantly enriched sucrose alpha-glucosidase activity, starch and sucrose metabolism, and carbon metabolism. It is to be noted that cucumber femaleness is positively correlated to the levels of glucose and sucrose, and the expressions of some genes involved in carbohydrate and energy metabolism are altered during low temperature-induced sex expression in cucumber^15,31^. These results suggest that blue light-induced femaleness in cucumber is potentially attributed to the enhancement in the photosynthetic processess and sugar pathway.

Notably, we found that an *MYB* transcription factor (Csa7G170600) was significantly down-regulated by the blue-light exposure (Table 6). Previous studies have shown that *CsGAMYB1* is predominantly expressed in the male-specific organs during cucumber flower development and regulates cucumber sex expression via an ET-independent pathway^36^. In addition, knockdown of *CsGAMYB1* results in a decreased ratio of nodes with male to female flowers. In the present study, blue light treatment down-regulated an *MYB* transcription factor and increased nodes with female flowers (Table 1 and Table 6). Blue light potentially inhibits the synthesis and transduction of GA, and a decreased GA content in the shoot apex might down-regulate *MYB* expression (Fig. 5 and Table 6). Thus, we propose that blue light stimulates female floral sex expression probably by modulating MYB via an ET-independent pathway^36^.

Another transcription factor, *AGL27*, which encodes an agamous-like MADS-box protein was down-regulated by the blue light treatment (Table 6). Previous reports demonstrated that members of the MADS-box family genes could control floral development and regulate the sexual development in cucumber^37^. In *Arabidopsis*, GA can induce the expression of an *Agamous-like MADS-box* gene, which is involved in flower development^38^. However, a recent report indicated that GA suppresses pistil development by inhibiting the expression of a *MADS-box* family gene *CAG2*, which eventually facilitates the development of male flowers^12^. Our data showed that blue light suppressed GA expression and influenced the expression of an *Agamous-like MADS-box* gene (*AGL27*), leading to an increased number of female flowers, suggesting that different MADS-box protein may have different roles in pistil development.

In summary, we found that among various light quality treatments, such as blue, green, red, and white light exposure on cucumber plants, BL-treated cucumber plants exhibited the highest female flowers in the first 20 nodes and the lowest first node of female flower. Transcriptome analysis, qRT-PCR and hormone qualification revealed that the blue light-induced female floral sex expression was mediated through hormone-related pathway mainly via decreased GA accumulation and its coordinated interaction with other hormones, the regulation of photosynthesis and starch, sucrose metabolism and transcription factor probably via an ET-independent pathway. This study lays a foundation for further exploring the molecular basis of blue light quality-induced sex expression and provides clues for breeding cucumber varieties with higher female sex differentiation trait and early maturity.

## Materials and Methods

### Plant materials and growth conditions

The monoecious cucumber (*Cucumis sativus* L.) cultivar Jinyan 4 seeds (obtained from Tianjin Cucumber Institute, Tianjin, China) were sown in 23 cm diameter-plastic pots containing a peat–vermiculite mixture (2:1, v/v). The pots were placed in a temperature-controlled greenhouse with a 12 h/12 h photoperiod and 25 °C/18 °C (day/night) temperatures. Seven days after germination, the seedlings were thinned to keep one healthy seedlings per pot, and fertilized once a week with Hoagland’s nutrient solution.

Seedlings (10 days after sowing) were exposed to different light qualities, such as red light (R, λ_red_ = 660 ± 5 nm), blue light (B, λ_blue_ = 465 ± 5 nm), green light (G, λ_green_ = 522 ± 5 nm), and white light (W, as the control), all of which were supplied from light-emitting diodes (LEDs, 10 W, Huizhou Kedao Technology Co. LTD, China). The intensity of light was set at 200 µmol·m^−2^·s^−1^ photosynthetic photon flux density (PPFD) at the level of canopy. Plant exposure to different qualities of lights existed for 20 days until the plants attained the 4-leaf stage.

Afterwards, the shoot apexes containing immature leaves shorter than 2 cm in length were sampled at 13:00 hr for RNA isolation and different biochemical analyses^15,16^. Samples were immediately frozen in liquid nitrogen and stored in refrigerator at −80 °C. After the light quality treatments, plants were transferred to a greenhouse in the practice base of Jiangxi Agriculture University, Nanchang, China (115°83′ E, 28°76′ N). A total of 20 nodes from each plant on the main stem were investigated to calculate the percentage of nodes with female flowers.

### Quantification of endogenous IAA and GA_3_ and chlorophyll content

To analyze the IAA and GA_3_ concentration, 0.5 g of frozen shoot apex sample was extracted in 4 mL of 80% methanol (v/v) with 1 mM 2,6-di-*t*-butyl-*p*-cresol. The homogenate was incubated at 4 °C for 4 h in the dark. After centrifugation for 20 min at 1000 g, crude extract supernatants were filtered through Sep-Pak C18 cartridge (Millipore, Milford, MA, USA) and dried under a stream of N_2_ gas. Dried samples were resuspended in 5 mL of 10% elution buffer (v/v) methanol in 50 mM Tris, pH 8.1, 1 mM MgCl_2_ and 150 mM NaCl. The concentrations of IAA and GA_3_ were quantified colorimetrically using a Multimode Plate Reader Label-free System (Perkin Elmer, Wellesley, MA, USA).

The shoot apex sample as well as the upper leaves were used for the quantification of chlorophyll (Chl a and Chl b) and carotenoids. The pigments were extracted in 80% acetone and the contents were determined spectrophotometrically according to the methods described previously^39^.

### RNA isolation and transcriptome sequencing

Total RNA was extracted from the blue and white light-exposed shoot apex samples using Trizol reagents (Invitrogen, Carlsbad, CA, USA) according to the manufacturer’s instruction. Three biological replicates were sequenced for each treatment and at least four plants were pooled for each biological replicate. The enrichment of mRNA, fragment interruption, addition of adapters, size selection, PCR amplification, and RNA-Seq were carried out at Beijing Novogene Bioinformatics Technology Co. Ltd (Beijing, China). Six biological samples from two different treatments were sequenced on the Illumina HiSeq X Ten platform and paired-end reads were generated for transcriptome sequencing.

### Transcriptome profile analysis

The raw reads generated from the sequencing machines were cleaned by discarding the adaptor sequences and low-quality reads, and by filtering the reads with an unknown nucleotide percentage greater than 5%. All following analyses were based on clean, high-quality data. The clean reads were aligned to reference genome sequences of the cucumber genome database (http://cucurbitgenomics.org/organism/2)^40^ using TopHat (v2.0.12) with default parameters.

To identify genes regulated by blue light compared with white light, *P* value ≤ 0.05 and the absolute value of log_2_(Fold change) with FPKM (fragments per kb per million reads) ≥1 were accepted as the thresholds for significantly differential expression. For pathway enrichment analysis, KOBAS software was used to test the statistical enrichment of DEGs in KEGG (Kyoto Encyclopedia of Genes and Genomes) pathways^41^. Pathway annotations of RNA-Seq genes were downloaded from KEGG. GO was performed using the GOseq R package (Release 2.12) based on Wallenius non-central hyper-geometric distribution to identify which DEGs were significantly enriched in GO terms. The GO annotations were functionally classified using the WEGO software for gene function distributions^42^.

### Quantitative real-time PCR (qRT-PCR)

Four genes involved in GA and ET biosynthesis and signaling pathways were selected to determine their expression under the blue light and white light treatments. And another 11 DEGs were randomly selected to confirm the expression level of RNA-Seq results using qRT-PCR according to protocols described previously. The cycle threshold values (C_T_) were determined and the relative fold differences were calculated by the 2^−ΔΔCt^ method^43^, and the cucumber *actin* gene (AB698859) was used as an internal control. The gene expression analysis for each treatment was performed with three biological replicates with three technical replicates. The sequences of gene-specific primers were shown in Supplementary Table S3.

### Statistical analysis

All data were analyzed using one-way analysis of variance (ANOVA) followed by Duncan’s multiple range test that compared the mean differences at *P* < 0.05. For the determination of sex expression of cucumber, 10 plants were used as a replicate for each treatment. There are four replicates for each treatment.

## Electronic supplementary material

Additional Information
