# Supplementary material for: Transcriptomic insights into the blue light-induced female floral sex expression in cucumber (Cucumis sativus L.)
Source: Sci Rep. 2018 Sep 24;8:14261. doi: 10.1038/s41598-018-32632-7 (PMC6155147; doi:10.1038/s41598-018-32632-7)
Supplement: Supplementary file 1 — Additional Information [file 41598_2018_32632_MOESM1_ESM.docx]

**Transcriptomic insights into** **the blue light**-**induced female floral sex expression in cucumber (*Cucumis sativus*** **L.)**

Yong Zhou^#1^, Golam Jalal Ahammed^#2^, Qiang Wang^1^, Chaoqun Wu^1^, Chunpeng Wan^1^, Youxin Yang^1^*

^1^ Jiangxi Key Laboratory of Crop Physiology, Ecology and Genetic Breeding, College of Agronomy/College of Science, Jiangxi Agricultural University, Nanchang 330045, China;

^2^College of Forestry, Henan University of Science and Technology, Luoyang 471023, China;

^#^These authors contributed equally to this work.

Correspondence and requests for materials should be addressed to Y.X.Y. (E-mail: [yangyouxin@jxau.edu.cn](mailto:yangyouxin@jxau.edu.cn)).

**Supplementary Table S1.** List of DEGs between blue and white light.

| Gene_ID | annotation | Fold change | pval |
| --- | --- | --- | --- |
| Csa3G097540 | Sesquiterpene synthase | 15.61 | 0.00 |
| NA | hypothetical protein VITISV_007304 | 7.52 | 0.00 |
| NA | uncharacterized LOC105436366 | 6.75 | 0.00 |
| Csa1G618390 | Cytochrome P450 | 6.54 | 0.00 |
| Csa6G085120 | Hfr-2-like protein | 6.39 | 0.02 |
| Csa5G605030 | Putative phytochrome kinase substrate | 6.07 | 0.01 |
| Csa1G065390 | Receptor-like protein kinase | 4.28 | 0.00 |
| Csa6G439940 | Calcium-dependent protein kinase | 3.96 | 0.00 |
| Csa2G415500 | MLO-like protein | 3.95 | 0.02 |
| Csa6G452010 | GDSL esterase/lipase | 3.94 | 0.00 |
| Csa6G476630 | Probable carboxylesterase 15 | 3.83 | 0.02 |
| Csa1G294090 | Ice binding protein, putative | 3.72 | 0.01 |
| Csa6G007450 | UDP-glucosyltransferase | 3.62 | 0.00 |
| Csa7G351890 | Quinone oxidoreductase | 3.49 | 0.00 |
| Csa7G064070 | Unknown protein | 3.40 | 0.00 |
| Csa1G145860 | Fatty acyl-CoA reductase 3 | 3.39 | 0.00 |
| Csa5G610430 | Auxin-responsive protein | 3.26 | 0.00 |
| Csa6G452640 | Soft fertilization envelope protein | 3.14 | 0.00 |
| Csa7G009100 | Auxin-induced SAUR-like protein | 3.06 | 0.03 |
| Csa5G517150 | Probable receptor-like protein kinase | 3.04 | 0.02 |
| Csa2G000730 | Acid phosphatase-like protein | 3.03 | 0.00 |
| Csa1G108270 | Unknown protein | 2.96 | 0.00 |
| Csa4G627100 | Gamma-glutamyl hydrolase | 2.93 | 0.03 |
| Csa3G040850 | Sesquiterpene synthase | 2.80 | 0.01 |
| Csa3G145780 | Putative early light induced protein 2 | 2.71 | 0.01 |
| Csa6G504720 | Cytochrome P450 | 2.54 | 0.04 |
| Csa4G293290 | Plant-specific domain TIGR01589 family protein | 2.47 | 0.00 |
| Csa5G622440 | Unknown protein | 2.45 | 0.02 |
| Csa6G419520 | Unknown protein | 2.42 | 0.03 |
| Csa5G292250 | Similarity to DNA helicase | 2.40 | 0.04 |
| NA | WAT1-related protein At5g64700-like | 2.34 | 0.01 |
| Csa6G421640 | Putative tyrosine-protein phosphatase | 2.32 | 0.01 |
| Csa6G077410 | Glycine-rich protein-like | 2.28 | 0.00 |
| Csa6G351370 | Gibberellin 20-oxidase | 2.27 | 0.05 |
| Csa1G600130 | Pheophorbide A oxygenase | 2.22 | 0.02 |
| Csa6G085110 | Hfr-2-like protein | 2.22 | 0.02 |
| Csa3G813300 | Ovarian cancer-associated gene 2 protein-like protein | 2.18 | 0.02 |
| Csa6G363010 | Ankyrin repeat family protein | 2.17 | 0.04 |
| Csa3G081380 | Unknown protein | 2.15 | 0.00 |
| NA | Unknown protein | 2.14 | 0.01 |
| Csa4G016490 | Anthranilate N-benzoyltransferase protein | 2.13 | 0.00 |
| Csa5G577430 | Protein CHUP1 | 2.13 | 0.05 |
| NA | probable linoleate 9S-lipoxygenase 5 | 2.12 | 0.00 |
| Csa1G524640 | Cytochrome P450 | 2.11 | 0.00 |
| Csa3G844890 | Knotted 1-binding protein 36 | 2.09 | 0.04 |
| Csa2G361840 | Cytochrome P450, putative | 2.07 | 0.01 |
| Csa4G003670 | Cp12 domain-containing protein 1 | 2.05 | 0.05 |
| Csa7G007820 | NADP-dependent D-sorbitol-6-phosphate dehydrogenase | 2.05 | 0.01 |
| Csa2G437130 | Major pollen allergen Bet v 1-L | 2.02 | 0.03 |
| Csa2G057080 | CONSTANS-like protein | 2.00 | 0.00 |
| Csa1G064730 | 1-aminocyclopropane-1-carboxylate oxidase 4 | 1.99 | 0.00 |
| Csa1G542430 | DNA primase/helicase | 2.02 | 0.05 |
| Csa5G598600 | Ethylene-responsive transcription factor | 0.49 | 0.03 |
| Csa7G428990 | Beta-glucosidase | 0.48 | 0.00 |
| Csa2G416220 | Cellulose synthase-like protein | 0.47 | 0.00 |
| Csa2G172480 | Cyclopropane-fatty-acyl-phospholipid synthase, putative | 0.47 | 0.01 |
| Csa7G452270 | Receptor-like protein kinase | 0.47 | 0.01 |
| Csa6G501330 | Flavonoid 3-hydroxylase, putative | 0.47 | 0.05 |
| Csa4G124910 | Hd1 | 0.45 | 0.00 |
| Csa3G829250 | Peroxidase | 0.45 | 0.02 |
| Csa4G625060 | Unknown protein | 0.44 | 0.00 |
| Csa2G176690 | Proline-rich protein | 0.43 | 0.04 |
| Csa7G170600 | MYB transcription factor | 0.42 | 0.04 |
| NA | periaxin-like | 0.41 | 0.00 |
| Csa6G095280 | Agamous-like MADS-box protein AGL27 | 0.39 | 0.00 |
| Csa6G012820 | Laccase, putative | 0.39 | 0.01 |
| Csa1G574820 | Cysteine synthase | 0.38 | 0.01 |
| Csa5G606310 | NAC domain-containing protein, putative | 0.35 | 0.00 |
| NA | hypothetical protein VITISV_007304 | 0.35 | 0.00 |
| Csa6G404120 | Probable peptide/nitrate transporter | 0.32 | 0.04 |
| Csa7G413380 | Gibberellin 2-oxidase, putative | 0.29 | 0.01 |
| Csa5G175870 | LOB domain-containing protein | 0.25 | 0.04 |
| Csa1G649550 | Cytochrome P450 | 0.23 | 0.00 |
| Csa7G363030 | Putative uncharacterized protein F3A4.230 | 0.08 | 0.01 |

**Supplementary Table S2.** qRT-PCR confirmation of select transcriptions identified by RNA-seq. Relative expression levels of selected genes in the treatment of white and blue light. The data of qRT-PCR are means of three independent biological, three technical replicates.

| **Gene ID** | **Description** | **RNA-Seq**  **(fold- change)** | **qRT-PCR**  **(fold- change)** |
| --- | --- | --- | --- |
| Csa1G064730 | AC04 | 2 | 1.6±0.02 |
| Csa7G413380 | Gibberellin 2-oxidase | 0.29 | 0.49±0.01 |
| Csa1G524640 | Abscisic acid 8'-hydroxylase | 2.11 | 1.7±0.01 |
| Csa5G610430 | Auxin-responsive protein, IAA32 | 3.25 | 4.73±0.01 |
| Csa6G439940 | Calcium-dependent protein kinase | 3.96 | 4.31±0.63 |
| Csa1G649550 | Cytochrome P450 | 0.23 | 0.24±0.03 |
| Csa4G124910 | Hd1 | 0.45 | 0.42±0.03 |
| Csa5G175870 | LOB domain-containing protein | 0.25 | 0.43±0.01 |
| Csa5G606310 | NAC transcription factor 29-like | 0.35 | 0.38±0.02 |
| Csa6G404120 | Probable peptide/nitrate transporter | 0.32 | 0.26±0.01 |
| Csa3G097540 | Sesquiterpene synthase | 15.61 | 9.35±0.98 |

**Supplementary Table S3**. primers used for qRT-PCR

| gene annotation | gene ID | Forwards primer | Reverse primer |
| --- | --- | --- | --- |
| *Actin* | AB698859 | GGAACTTTGGTGGTCGTGAC | TTCGGCAGTGGTGGTGAA |
| *ACS2* | Csa1M580750 | GTGCCACTGCTGCCAATG | TTCACTCCTGTTCTCCATCTCAA |
| *ETR1-F* | Csa2M070880 | CTCTTCATCAGCAGAATCCAGTG | ACGAACAGCAACAACCTCTC |
| *GA20ox* | Csa3M179110 | CGAGAAGCCAGAGTTGACATTAG | AGGAGTCTGTTGTTGGAGGAA |
| *GA3ox* | Csa7M434970 | CACTCCATTGAATCCTCTGCTAAT | GCCTTCCGACCACATACG |
| *AC04* | Csa1G064730 | CCTCCGCCACTTCTATTACAC | CCGCATATTCTTCCATTGTTGAC |
| *Abscisic acid 8'-hydroxylase* | Csa1G524640 | GCAATGAAGGCAAGGAAGGT | ACCACCACCGCTGAGTAG |
| *Cytochrome P450* | Csa1G649550 | GCACCTTCATCGTTAAGTTCCT | CCTCACAAGCCACATTCCAT |
| *Sesquiterpene synthase* | Csa3G097540 | CCCACAAGTTTGAACAAGAAAGG | ACACGCATTAGAACAGGCAAT |
| *Hd1* | Csa4G124910 | ATCATCATCACGAACAGCAACA | TGAGGAAGCCATTGTAGGAGAA |
| *LOB domain-containing protein* | Csa5G175870 | GTTCACCATTGCCCATAGAGTT | CTTGTAGTTCGCTCACTTGCT |
| *NAC transcription factor 29-like* | Csa5G606310 | CCAAGCCACCTCCAAACC | GCACTGTAAATCGCCTTATCTGT |
| *Auxin-responsive protein, IAA32* | Csa5G610430 | GATGGTGTCATTGTTGGTAGGAA | AACCTCAGTCCAGATTCACATTG |
| *Probable peptide/nitrate transporter* | Csa6G404120 | ATAGAACAAGCCGCCACAAT | CGAGGATGATGATGAATAGGACAG |
| *Calcium-dependent protein kinase* | Csa6G439940 | CTGGTCATAACATTTCGGCTCAT | CTCTTCGCACATCCTCAATATCG |
| *Gibberellin 2-oxidase* | Csa7G413380 | CAAGTCCATCGGCTCCAAC | GGTCAAGAATCTCGCATCCTAAG |


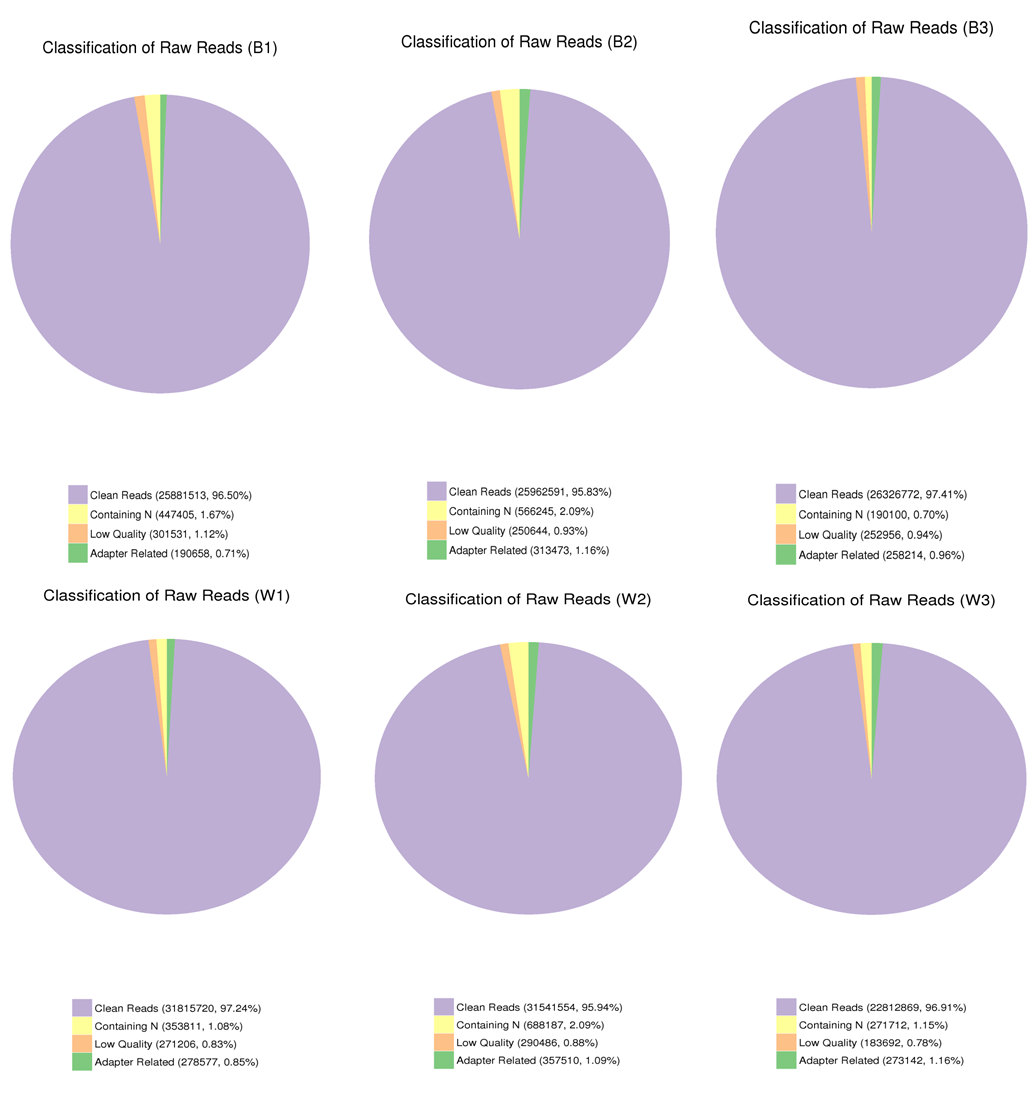


**Supplementary Figure S1.** The quality of the raw reads


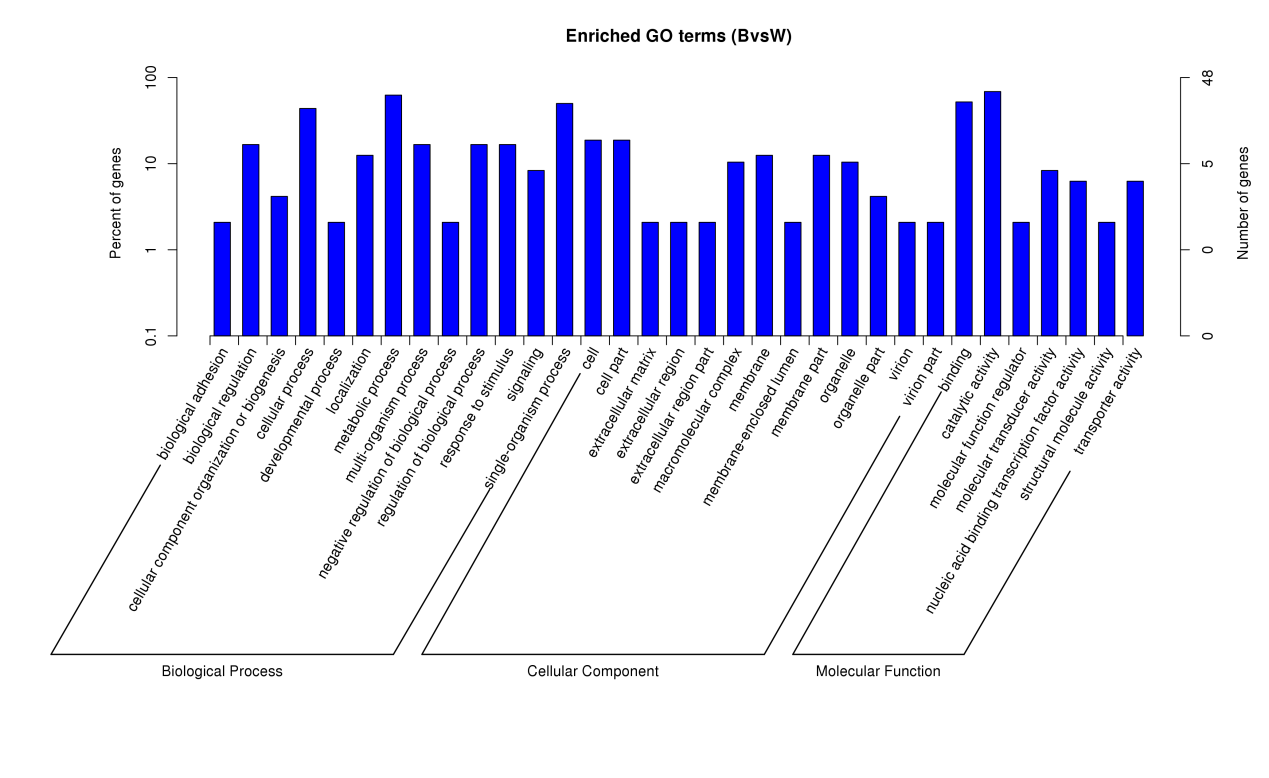


**Supplementary Figure S2.** GO annotation of DEGs.
